# Supplementary material for: Activation of the Pseudomonas aeruginosa glycerol regulon reduces antibiotic susceptibility and modulates virulence phenotypes
Source: Infect Immun. 2025 Sep 22;93(10):e00410-25. doi: 10.1128/iai.00410-25 (PMC12519775; doi:10.1128/iai.00410-25)
Supplement: Fig. S1 — Role of glpR in aminoglycoside tolerance. [file iai.00410-25-s0001.pdf]

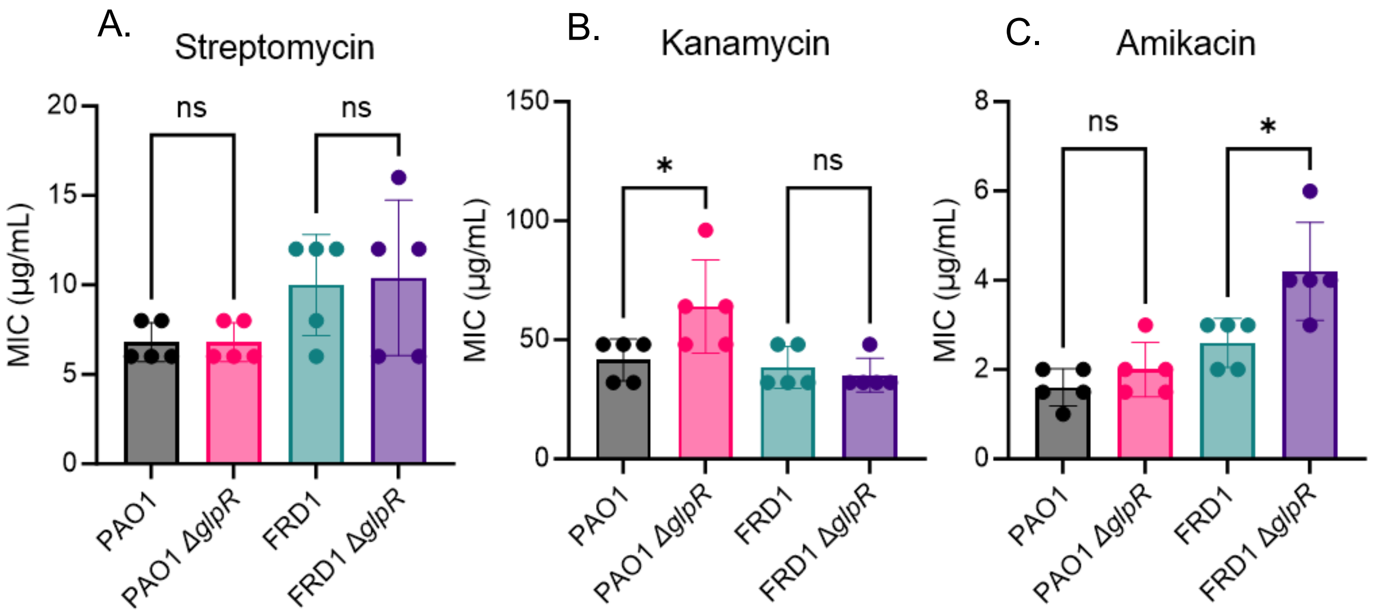

Figure S1: Role of *glpR* in aminoglycoside tolerance. Determination of MIC for three drug classes with *glpR* knockout. All MIC assays were conducted using Mueller Hinton Agar with lawns grown for 24 hours, and MIC strips provided by Liofilchem. MIC measurements for strains exposed to a gradient of A). streptomycin B). kanamycin C). amikacin *Statistical significance assessed by t-test. *P<0.05, ns=not significant
